# Supplementary material for: Synergy between serum amyloid A and secretory phospholipase A2
Source: eLife. 2019 May 21;8:e46630. doi: 10.7554/eLife.46630 (PMC6557629; doi:10.7554/eLife.46630)
Supplement: Figure 1—source data 1. [file elife-46630-fig1-data1.docx]

Figure 1C was plotted as follows:

A – X-axis

D and B are Y-axis values.

E and F – error bars
