# Supplementary material for: Synergy between serum amyloid A and secretory phospholipase A2
Source: eLife. 2019 May 21;8:e46630. doi: 10.7554/eLife.46630 (PMC6557629; doi:10.7554/eLife.46630)
Supplement: Figure 2—source data 1. [file elife-46630-fig2-data1.docx]

Figure 2A – Source data 2A

Figure 2B – source data 2B

Figure 2C- source data 2C

Figure 2D-source data 2D

1. X-axis
2. Y-axis

E-error
