# Supplementary material for: Synergy between serum amyloid A and secretory phospholipase A2
Source: eLife. 2019 May 21;8:e46630. doi: 10.7554/eLife.46630 (PMC6557629; doi:10.7554/eLife.46630)
Supplement: Figure 6—source data 1. [file elife-46630-fig6-data1.docx]

Figure 6 – source data 6

1. X-axis

B,C and D- Y-axis

E,F and G - error
