# Supplementary material for: Synergy between serum amyloid A and secretory phospholipase A2
Source: eLife. 2019 May 21;8:e46630. doi: 10.7554/eLife.46630 (PMC6557629; doi:10.7554/eLife.46630)
Supplement: Figure 6—figure supplement 1—source data 1. [file elife-46630-fig6-figsupp1-data1.docx]

Figure 6 – Figure supplement 1 - source data Figure supplement 1

1. X – axis

B,C, and E – Y-axis

F,D, and G - error
