# Supplementary material for: Synergy between serum amyloid A and secretory phospholipase A2
Source: eLife. 2019 May 21;8:e46630. doi: 10.7554/eLife.46630 (PMC6557629; doi:10.7554/eLife.46630)
Supplement: Figure 6—figure supplement 2—source data 1. [file elife-46630-fig6-figsupp2-data1.docx]

Figure S6 – Figure supplement 2 - source data figure supplement 2

1. X-axis

B, C, and E – Y-axis

F,D, and G - error
